# Rational Proteomic Analysis of a New Domesticated *Klebsiella pneumoniae* x546 Producing 1,3-Propanediol

**DOI:** 10.3389/fmicb.2021.770109

**Published:** 2021-11-26

**Authors:** Xin Wang, Lin Zhang, Hong Chen, Pan Wang, Ying Yin, Jiaqi Jin, Jianwei Xu, Jianping Wen

**Affiliations:** ^1^Key Laboratory of Systems Bioengineering (Ministry of Education), Tianjin University, Tianjin, China; ^2^SynBio Research Platform, Collaborative Innovation Center of Chemical Science and Engineering (Tianjin), School of Chemical Engineering and Technology, Tianjin University, Tianjin, China; ^3^Department of Chemistry, National University of Singapore, Singapore, Singapore; ^4^Institute of Materials Research and Engineering, Singapore, Singapore; ^5^Dalian Petrochemical Research Institute of Sinopec, Dalian, China

**Keywords:** *Klebsiella pneumoniae*, 1, 3-propanediol production, betaine, Na^+^ pH neutralizer, proteomics

## Abstract

In order to improve the capability of *Klebsiella pneumoniae* to produce an important chemical raw material, 1,3-propanediol (1,3-PDO), a new type of *K. pneumoniae* x546 was obtained by glycerol acclimation and subsequently was used to produce 1,3-PDO. Under the control of pH value using Na^+^ pH neutralizer, the 1,3-PDO yield of *K. pneumoniae* x546 in a 7.5-L fermenter was 69.35 g/L, which was 1.5-fold higher than the original strain (45.91 g/L). After the addition of betaine, the yield of 1,3-PDO reached up to 74.44 g/L at 24 h, which was 40% shorter than the original fermentation time of 40 h. To study the potential mechanism of the production improvement of 1,3-PDO, the Tandem Mass Tags (TMT) technology was applied to investigate the production of 1,3-PDO in *K. pneumoniae*. Compared with the control group, 170 up-regulated proteins and 291 down-regulated proteins were identified. Through Gene Ontology and Kyoto Encyclopedia of Genes and Genomes pathway analysis, it was found that some proteins [such as homoserine kinase (ThrB), phosphoribosylglycinamide formyltransferase (PurT), phosphoribosylaminoimidazolesuccinocarboxamide synthase (PurC), etc.] were involved in the fermentation process, whereas some other proteins (such as ProX, ProW, ProV, etc.) played a significant role after the addition of betaine. Moreover, combined with the metabolic network of *K. pneumoniae* during 1,3-PDO, the proteins in the biosynthesis of 1,3-PDO [such as DhaD, DhaK, lactate dehydrogenase (LDH), BudC, etc.] were analyzed. The process of 1,3-PDO production in *K. pneumoniae* was explained from the perspective of proteome for the first time, which provided a theoretical basis for genetic engineering modification to improve the yield of 1,3-PDO. Because of the use of Na^+^ pH neutralizer in the fermentation, the subsequent environmental pollution treatment cost was greatly reduced, showing high potential for industry application in the future.

## Introduction

The rise of the biodiesel industry leads to the overproduction of glycerol as a by-product, which now threatens the economic feasibility of the industry (Pan et al., 2019; Kim et al., 2020). This situation has prompted scientists to explore the utilization of glycerol as a carbon source to produce 1,3-propanediol (1,3-PDO), which is a precursor of some important commercial polymers such as polyester and polyurethane (Zhou et al., 2019b; Bao et al., 2020; Chen et al., 2020). 1,3-PDO can be produced by chemical synthesis or biosynthesis using *Klebsiella pneumoniae*. Because of its relatively high yield and low environmental pollution, *K. pneumoniae* is preferable to be used in 1,3-PDO production (Li et al., 2019; Mitrea and Vodnar, 2019; Zabed et al., 2019). Researchers have adopted several strategies such as the domestication method, genetic modification, medium optimization, and other methods to significantly increase the output of 1,3-PDO (Zhang et al., 2018; Lee et al., 2019; Ma et al., 2019; Zhou et al., 2019a). However, the industrial-scale production of 1,3-PDO using bacteria is still limited by low efficiency, which seriously hinders the competitiveness of the process (Dexter Tam et al., 2019; Guo et al., 2019; Park et al., 2019).

The proteomic analysis of protein expression patterns under experimental conditions can provide sufficient information on the function and the regulation of metabolic networks, which is important in the reasonable and purposeful exploration of genome and proteome datasets for the pathway analysis of actual biological processes in post–genome research. The Tandem Mass Tags (TMT) technology was one of the most powerful analytical methods with the highest flux, the smallest systematic error, and the most powerful function (Sogame et al., 2014). It could provide more accurate digital signals, higher detection fluxes, and wider detection ranges. A more detailed understanding of the metabolic pathway of *K. pneumoniae* and other species could help to provide a better way to promote the transformation of glycerol into 1,3-PDO in this system. Therefore, it is necessary to apply the TMT technology to the study of 1,3-PDO production by *K. pneumoniae*.

Ca^2+^ salt as a divalent cation can reduce the drastic changes in the activities of various intracellular dehydrogenases in the oxidation pathway, adjust and maintain the intracellular redox pressure, shift the metabolic flow to 1,3-PDO synthesis, and reduce the types of by-products caused by metabolic disorders. Therefore, Ca^2+^ salts have been commonly used as a pH neutralizer in industry (Nakano et al., 2012; Zhang et al., 2016; Tee et al., 2017). However, various Ca^2+^ salt precipitates were formed when using Ca^2+^ neutralizer at the bottom of the fermentation tank, which not only increases the cost of the subsequent product purification, but also causes significant environmental pollution. Considering the environmental pressure caused by the utilization of Ca^2+^ neutralizer, it is critical that Ca^2+^ pH neutralizer be replaced with a new neutralizer without compromising with production efficiency, so as to make the 1,3-PDO bioproduction more environment-friendly. Na^+^ pH neutralizer can reduce the solid pollutants produced after fermentation reaction. As Na_2_CO_3_ can be synthesized by a chemical method from the electrolysis of high salt wastewater containing Na^+^ and re-extracted for reuse (Shin et al., 2011; Simon et al., 2014), the use of Na_2_CO_3_ as a pH adjuster in fermentation would promote a new industrial recycling. However, the use of Na^+^ in fermentation leads to an increase in osmotic pressure, thereby restricting the yield of 1,3-PDO (Glaasker et al., 1998; Guerzoni et al., 2001), whereas betaine can slow down the effect of salt stress (Hussain et al., 2020). It can maintain the balance of osmotic pressure inside and outside, thereby maintaining the normal physiological function of the cell (Louesdon et al., 2014). Moreover, the betaine may have an effect on fermentation under Na^+^ conduction.

In this study, Na_2_CO_3_ was used as the pH neutralizer in fermentation, and betaine was added to alleviate the high osmotic pressure caused by excessive Na^+^, which would significantly enhance the yield of 1,3-PDO. The 1,3-PDO production further increased to 74.44 g/L and shortened the fermentation time from 40 to 24 h. TMT was used to study the mechanism effects of the introduction of the Na^+^ neutralizer and betaine on the yield of 1,3-PDO during the fermentation. This is the first comprehensive investigation of TMT analysis for the production of 1,3-PDO by *K. pneumoniae* x546, and the results will provide new insights on enhancing the production of 1,3-PDO (genes, proteins, and metabolites), as well as the subsequent industrial strain transformation and process optimization.

## Materials and Methods

### Strains, Media, and Cultivations

*Klebsiella pneumoniae* American Type Culture Collection (ATCC) 15380 was purchased from the ATCC. Following the previously published adaptive laboratory evolution (Willke and Vorlop, 2008; Gungormusler et al., 2011; Raghunandan et al., 2014), *K. pneumoniae* x546 (domesticated strain with 120–20 g/L glycerol: the strain was first domesticated with a concentration of 120 g/L glycerol and then returned to a concentration of 20 g/L glycerol for domestication) could be obtained (the details could be seen in Supplementary File 1). The seed and solid medium (pH 7.0) contained 40 g/L (60, 80, 100, 120, 140, 100–20, 120–20, and 140–20 g/L) glycerol, 4.08 g/L NH_4_Cl, 0.57 g/L KCl, 0.95 g/L NaH_2_PO_4_⋅2H_2_O, 0.28 g/L Na_2_SO_4_, 0.25 g/L MgCl_2_⋅6H_2_O, 0.38 g/L citric acid, 0.95 g/L yeast extract, 0.15 g/L Vc, and 4 mL of nutrient solution. Nutrient solution contained 0.035 g/L Na_2_MoO_4_, 0.029 g/L ZnCl_2_, 0.29 g/L CoCl_2_⋅6H_2_O, 0.148 g/L MgSO_4_⋅7H_2_O, 0.033 g/L NiCl_2_⋅6H_2_O, and 1.0 mL HCl. The *K. pneumoniae* was domesticated with 40 g/L (60, 80, 100, 120, 140, 100–20, 120–20, and 140–20 g/L), and glycerol was labeled G40, G60 G80, G120, G140, G100–20, G120–20, and G140–20 (Liang et al., 2018).

The production medium was a little different from the seed medium, which contained 40 g/L glycerol, 6.17 g/L NH_4_Cl, 0.86 g/L KCl, 1.40 g/L NaH_2_PO_4_⋅2H_2_O, 0.32 g/L Na_2_SO_4_, 0.3 g/L MgCl_2_⋅6H_2_O, 1.06 g/L citric acid, 1.15 g/L yeast extract, 0.25 g/L betaine, 0.11 g/L Vc, 0.23 g/L C_5_H_11_NO_2_, and 5 mL of nutrient solution. Nutrient solution contained 5.4 g/L FeCl_3_⋅6H_2_O, 0.004 g/L Na_2_MoO_4_, 0.04 g/L ZnCl_2_, 0.17 g/L MnCl_2_⋅4H_2_O, 0.47 g/L CoCl_2_⋅6H_2_O, 0.06 g/L H_3_BO_4_, 0.68 g/L CuSO_4_⋅5H_2_O, and 1.0 mL HCl. The betaine was added only in the production medium for 7.5 L fermenter.

The seed was cultivated in 250-mL flask containing a 100-mL seed medium at 150 revolutions/min (rpm) for 8.5 h at 37°C. The production of 1,3-PDO was carried out in a 250-mL flask with 100 mL working volume at 150 rpm for 48 h at 37°C and in a 7.5-L BioFlo 110 fermenter (New Brunswick Scientific, Edison, NJ, United States) at 400 rpm for 40 h at 37°C after adding 770 g glycerol (with a final 5.4 L working volume). Three biological replicates were used for each fermentation experiment. The pH of the seed medium and fermentation medium was adjusted to 7.0 with 3.125 M Na_2_CO_3_ solution, respectively.

### Determination of 1,3-Propanediol and Glycerol Concentrations

The concentration of 1,3-PDO and glycerol was measured by an HPX-87H column (300 mm × 7.8 mm) (Bio-Rad, Palo Alto, CA, United States) with a differential refractive index detector (SFD GmbH, Schambeck, Germany); 5 mM H_2_SO_4_ was used as a mobile phase with a flow rate of 0.5 mL/min at a working temperature of 65°C.

### Total Protein Extraction

The samples were ground into a powder in liquid nitrogen. Then the powder was suspended in lysis buffer (1% sodium deoxycholate, 8 M urea). The mixture was allowed to settle at 4°C for 30 min during which the sample was vortexed every 5 min and treated by ultrasound at 40 kHz and 40 W for 2 min. After centrifugation at 16,000 rpm at 4°C for 30 min, the concentration of protein supernatant was determined by bicinchoninic acid (BCA) method by BCA Protein Assay Kit (Pierce, Thermo, United States). Protein quantification was performed according to the kit protocol (Chen et al., 2021).

### Protein Digestion and Tandem Mass Tags Labeling

Protein digestion was performed according to a standardized procedure, and the resulting peptide mixture was labeled using 10-plex TMT reagent (Thermo Fisher, Scientific). In brief, an aliquot of protein (100 μg) from each sample was mixed with 100 μL of the lysate. Then 10 mM TCEP was added, and the mixture was stored at 37°C for 60 min, followed by the addition of 40 mM iodoacetamide and the storage of the sample in the dark at room temperature for 40 min.

Sixfold volumes of cold acetone were added to precipitate the protein at −20°C for 4 h. After centrifuging at 10,000 rpm for 20 min at 4°C, the pellet was resuspended with 100 μL of 50 mM triethylammonium bicarbonate buffer. Trypsin was added at a trypsin-to-protein mass ratio of 1:50 and incubated at 37°C overnight. One unit of TMT reagent was thawed and reconstituted in 50 μL acetonitrile. After tagging for 2 h at room temperature, hydroxylamine was added to react with mixture for 15 min at room temperature (Dai et al., 2020).

In this work, the strains grown under control (fermentation of the strain domesticated at a glycerol concentration of 40 g/L without betaine) and optimal conditions (fermentation of the strain domesticated at a glycerol concentration of 120–20 g/L with betaine) at 10 h (each sample with two biological replicates) were collected by centrifugation (12,000 rpm, 10 min at 4°C) and frozen in liquid nitrogen, respectively. The samples were labeled as A1, A2, B1, and B2. Finally, all samples were pooled, desalted, and vacuum-dried for subsequent use. To verify the accuracy of proteomic data, quantitative real-time polymerase chain reaction (qRT-PCR) was also done (the details are shown in Supplementary File 1).

### Liquid Chromatography–Tandem Mass Spectrometry Analysis

Labeled peptides were analyzed by online nano flow liquid chromatography tandem mass spectrometry (MS/MS) using the 9RKFSG2_NCS-3500R system (Thermo Fisher Scientific) connected to the Q_Exactive HF-X system (Thermo Fisher Scientific) via a nanoelectrospray ion source. Briefly, a C18-reversed phase column (75 μm × 25 cm, Thermo Fisher Scientific) was equilibrated with solvent A (A: 2% acetonitrile and 0.1% formic acid) and solvent B (B: 80% acetonitrile and 0.1% formic acid). The peptides were eluted using the following gradient: 0–2 min, 0–3% B; 2–92 min, 5–25% B; 92–102 min, 25–45% B; 102–105 min, 45–100% B; 105–120 min, 100–0% B at a flow rate of 300 μL/min. The Q_Exactive HF-X was operated in the data-dependent acquisition mode to automatically switch between full scan MS and MS/MS acquisition. The survey of full scan MS spectra (m/z 350–1,500) was acquired in the Orbitrap with 70,000 resolutions. The top 20 most intense precursor ions were selected into the collision cell for fragmentation by higher-energy collision dissociation. The MS/MS resolution was set at 35,000 (at m/z 100), with the maximum fill time of 50 ms and a dynamic exclusion of 30 s (Wang et al., 2020a,b).

### Protein Identification

The RAW data files were analyzed by Proteome Discoverer 2.2 (Thermo Fisher Scientific) against the *K. pneumoniae* database^1^. The MS/MS search criteria were as follows: a mass tolerance of 20 ppm for MS and 0.02 Da for MS/MS tolerance, trypsin as the enzyme with two-missed cleavages allowed, carbamido methylation of cysteine and the TMT of the N-terminus and lysine side chains of peptides as fixed modification, and methionine oxidation as dynamic modifications, respectively. The false discovery rate for peptide identification was set at ≤0.01. A minimum of one unique peptide identification was used to support protein identification (Wang et al., 2020c).

### Statistical Analyses

The thresholds of fold change (FC) (>1.2 or <0.83) and *p* < 0.05 were used to identify differentially expressed proteins (DEPs). Annotation of all identified proteins was performed by Gene Ontology (GO)^2^ and Kyoto Encyclopedia of Genes and Genomes (KEGG) pathway^3^ analyses. DEPs were further used for GO and KEGG enrichment analysis. Protein–protein interaction analysis was performed using the String v10.5.

## Results and Discussion

### Comparison of the 1,3-Propanediol Production

**FIGURE 1:**
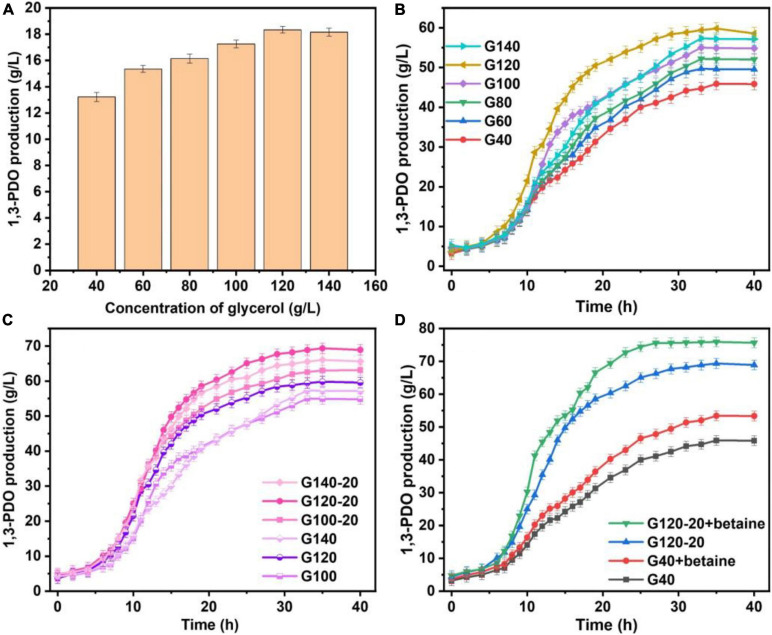
**(A)** Compa re the production of 1,3-PDO in the 250-mL shaker with glycerine domestication concentration at 40, 60, 80, 100, 120, and 140 g/L. **(B)** The comparison of the yield of 1,3-PDO in the 7.5-L fermentation tank with glycerine domestication concentration at 40, 60, 80, 100, 120, and 140 g/L. **(C)** The production 1,3-PDO in the 7.5-L fermentation tank with glycerine domestication concentration at 100, 100–20, 120, 120–20, 140, and 140–20 g/L. **(D)** The capacity of 1,3-PDO in the 7.5-L fermentation tank with glycerine domestication concentration at 40 g/L, 40 g/L +betaine, 120–20 and 120–20 g/L +betaine.

The yields of 1,3-PDO produced in 250-mL shaker by the original *K. pneumoniae* and the domesticated *K. pneumoniae* with and without betaine are summarized in Figure 1A. The 1,3-PDO production increased from G40 (13.22 g/L) to G120 (18.34 g/L), but decreased at G140. To further investigate the changes in yield during fermentation, the production of 1,3-PDO in the 7.5-L fermentation tank with glycerol domestication concentrations of 40, 60, 80, 100, 120, and 140 g/L were studied, and the results are shown in Figure 1B. The 1,3-PDO yields for glycerol domestication concentration from 40 to 120 g/L were 45.91, 49.71, 52.21, 55.02, 59.82, and 57.34 g/L, respectively. When the glycerol acclimation concentration increased to 140 g/L, the 1,3-PDO production decreased. With the increase in the domestication concentration of glycerol for the *K. pneumoniae*, the yield of 1,3-PDO decreased, which was consistent with the previous results (Colin et al., 2000; Yiqiang et al., 2007; Metsoviti et al., 2012; Raghunandan et al., 2014). When the concentration of glycerol increased, metabolism was inhibited, and 1,3-PDO production decreased. Therefore, G100, G120, and G140 were redomesticated under the concentration of 20 g/L glycerol. From Figure 1C, the yields of 1,3-PDO increased to 63.11, 69.35, and 66.03 g/L for G100–20, G120–20, and G140–20, respectively. After comparing G120–20 with G40, the yield of 1,3-PDO was improved by 51.06%. During the whole fermentation process, Na_2_CO_3_ was used as the pH neutralizer, which had an effect on the osmotic pressure of the fermentation liquid and the production yield of 1,3-PDO, so betaine as a fermentation medium was added (Fan et al., 2018). In Figure 1D, the 1,3-PDO yields of the G40 (+betaine) and G120–20 (+betaine) reached the 53.42 g/L and 75.92 g/L, respectively, which were higher than those without betaine, consistent with a previous report (Jantama et al., 2010). Thus, betaine could alleviate the osmotic pressure problem and ensure the activity of bacteria during fermentation. Compared to G120–20 (+betaine), which reached a yield of 74.44 g/L at 24 h, the 1,3-PDO production yield of G40 was very low (45.91 g/L) and the fermentation time was very long (40 h), further confirming that betaine could alleviate the increase in the osmotic pressure and counter-suppress the Na^+^ effect during fermentation. To explore the enhanced mechanism of 1,3-PDO production after the addition of Na_2_CO_3_ and betaine, G40 was used as a control group, and TMT was used to compare the differences in protein expression between the control and optimal G120–20 (+betaine).

### Protein Identification and Quantitation

After the G40 and G120–20 (+betaine) fermented broths were labeled by TMT, the primary and secondary mass spectra were analyzed statistically. With the help of the Protein Discoverer search library, a total of 3,284 proteins were identified from the four samples. Supplementary Figure 3A shows the number distribution of peptides contained in the identified proteins. For example, there were 521 proteins matched with one peptide. The length distribution of the identified peptides is shown in Supplementary Figure 3B. For instance, there were 1,860 proteins with a peptide length of eight amino acids. Most of the peptides had 5 to 20 amino acids after enzymatic hydrolysis, accounting for 83.87% of the total, which indicated that the enzymatic hydrolysis was sufficient, and the identification results were reliable. As shown in Supplementary Figure 3C, the molecular weight distribution of the identified proteins was determined, especially for these proteins with low molecular weights of less than 20 kDa. The molecular weights of most proteins were from 1 to 60 kDa, and 65 types of macromolecular proteins with molecular weights of more than 100 kDa were identified. Supplementary Figure 3D shows the coverage distribution of the identified proteins. The number of amino acids in the peptide was higher than the total number of amino acids in the protein. The identification results were more persuasive with the expansion of coverage distribution. The coverages of polypeptides with more than 10 and 20% of the identified proteins were 78.72 and 62.68%, respectively.

**FIGURE 2:**
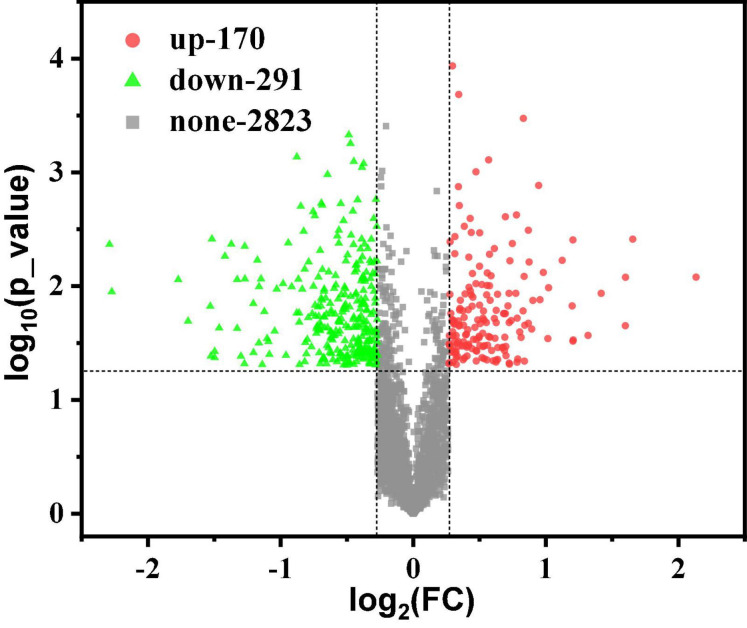
Scatter plot of volcano differences. A volcano plot between the G40 and the G120–20 (+betaine) whose proteins are shown. The horizontal axis is the value of log_2_(FC). The vertical axis is the –log_10_ (*p*-value). The red dots represent up-regulated proteins (FC > 1.2), and the green dots represent down-regulated proteins (FC < 0.83).

Usually, proteins with differences between G40 and G120–20 were determined based on the FC and the *p*-value. In this work, *p* < 0.05 indicates the difference among the groups. As shown in Figure 2, there were 3,284 proteins, including 170 up-regulated proteins (FC > 1.2) and 291 down-regulated proteins (FC < 0.83). These detected proteins were analyzed by GO term and KEGG pathway analysis to identify the biological functions of the differential proteins and the target proteins.

### The Analysis for Gene Ontology Term

**FIGURE 3:**
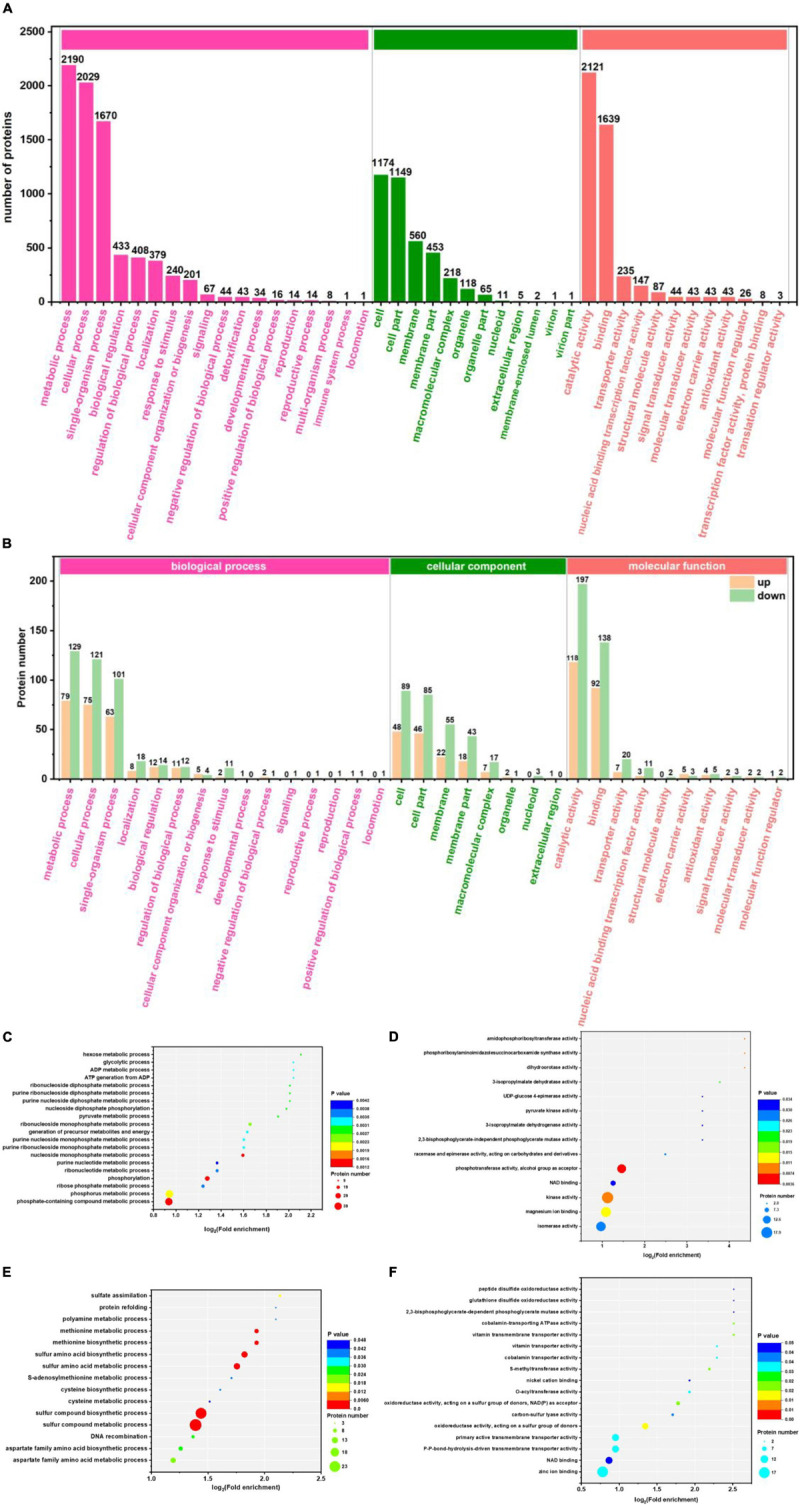
Gene Ontology (GO) classification and enrichment analysis of G40 and G120–20 (+betaine). **(A)** GO classification of 3,284 proteins. The ordinate on the left is –log_10_ (percent of proteins), and the label on the column is the number of proteins. **(B)** GO classification of 170-up and 291-down proteins. **(C)** The enrichment analysis of the BP (170-up proteins). **(D)** The enrichment analysis of the BP (291-down proteins). **(E)** The enrichment analysis of the MF (170-up proteins). **(F)** The enrichment analysis of the MF (291-down proteins). [The *p*-value (corrected) < 0.005].

With the GO database, genes and gene products can be classified and annotated as follows: cellular component (CC), molecular function (MF), and Biological Process (BP). It is a bioinformatics analysis tool (Zhong et al., 2019). Figures 3A,B show the level 2 of GO classification for 3,284 proteins and 461 differential proteins, respectively. For the 3,284 proteins: 2,190, 2,029, and 1,670 proteins were detected in metabolic process, the cellular process, and single organization process of BP, respectively. There were 1,174 and 1,149 proteins detected in cell and cell part of CC, respectively. There were 2,121 and 1,639 proteins detected in catalytic activity and binding of MF, respectively. As can be seen from Figure 3B, in BP: 115 up-regulated and 189 down-regulated differential proteins were detected in metabolic process; 104 up-regulated and 172 down-regulated differential proteins were detected in the cellular process; 85 up-regulated and 154 down-regulated proteins were detected in the single organization process. In CC, 52 up-regulated and 93 down-regulated differential proteins were detected in cell; 51 up-regulated proteins and 89 down-regulated proteins were detected in the cell part. In MF, 108 up-regulated and 192 down-regulated differential proteins were detected mainly in catalytic activity; 86 up-regulated and 139 down-regulated differential proteins were detected in binding. Compared with Figures 3A,B, the main functional area was the same, which indicated that these functions played an important role in the production of 1,3-PDO by *K. pneumoniae*. However, because of the excessive number of proteins, the enrichment of differentially abundant proteins requires further analysis.

**FIGURE 4:**
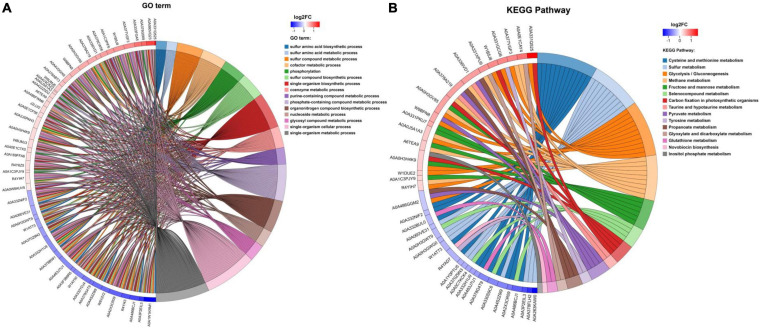
**(A)** Chord diagram of GO term enrichment. **(B)** Chord diagram of KEGG pathway enrichment. KEGG enrichment chord diagram shows the corresponding relationship among the target proteins, the annotation, and enrichment; the left is protein, and log_2_FC is displayed from top to bottom. When log_2_FC > 0, the larger the log_2_FC is, the greater the differential expression of up-regulated protein is; when log_2_FC < 0, the smaller the log_2_FC is, the greater the differential expression of down-regulated protein is. The closer the log_2_FC is to 0, the smaller the differential expression fold is. The right is KEGG pathway and the score of *z* score. When *z* score > 0, there are more up-regulated proteins than down-regulated proteins involved in this pathway, and this pathway is more likely to be activated. On the contrary, when *z* score < 0, the up-regulated proteins involved in this pathway are less than the down-regulated proteins, and this pathway is more likely to be inhibited. (The same analysis is applied to GO term).

Gene Ontology functional enrichment analysis can clarify the biological process, cell components, and molecular functions (Zhong et al., 2019). The enrichment of up-regulated and down-regulated proteins for BP is shown in Figure 3C and Table 1. Up-regulated proteins were related to the ribonucleoside monophosphate metabolic process, nucleoside monophosphate metabolic process, purine nucleoside monophosphate metabolic process, and so on. In MF (Figure 3E and Table 1), up-regulated proteins were related to phosphotransferase activity (alcohol group as acceptor). In BP (Figure 3D), down-regulated proteins were associated with sulfur compound metabolic process, sulfur compound biosynthetic process, sulfur amino acid biosynthetic process, sulfur amino acid metabolic process, methionine biosynthetic process, and methionine metabolic process. Finally, there were 41 up-regulated proteins and 19 down-regulated proteins after further analysis. Therefore, further analysis was needed through the chord diagram of GO term enrichment. As shown in Figure 4B, the most up-regulated proteins (1.23 ∼ 3.03-fold) were identified to be NAD-dependent glyceraldehyde-3-phosphate dehydrogenase (GAPDH), 4-hydroxythreonine-4-phosphate dehydrogenase (PdxA), phosphoenolpyruvate-dihydroxyacetone phosphotransferase (DhaL), ThrB, PurT, phosphoglycerate kinase (PGK), 2,3-bisphosphoglycerate-independent phosphoglycerate mutase (GpmI), PurC, and so on. PdxA, whose function is similar to the isocitrate dehydrogenase and isopropylmalate dehydrogenase, can contribute to the phosphotransacetylase (Pta) activity (Sivaraman et al., 2003; Xu et al., 2005). It has been reported that Pta plays a role in the reduction pathway of 1,3-PDO produced by *K. pneumoniae*. It is well known that amino acid metabolism plays an important role in the life of *K. pneumoniae*, which can balance the intracellular pH, generate energy, reduce power, and resist environmental pressures. With the increase in the ThrB, the more threonine is produced. The *K. pneumoniae* can use threonine as a nitrogen source (Reitzer, 2005), and the cavity near ADP is very suitable for homoserine binding, so it can improve the catalytic activity by stabilizing the transition state (Fan et al., 2009; Zhang et al., 2019) and contribute to the 1,3-PDO production. The ligation of amino and carboxylate groups of small molecule metabolites is catalyzed by the ATP-grasp superfamily, which is widespread across primary metabolic processes (Zhang et al., 2008). PurT is a member of the ATP-grasp superfamily, and PurC also has several structural elements in common. With the up-regulation of PurC, the expression levels of diverse proteins involved in purine and pyrimidine synthesis, carbon and energy metabolisms, iron uptake, proteolysis, protein secretion, and signal transduction can be improved. Purine can save energy from the beginning and the consumption of some amino acids (Yuan et al., 2013). As a key enzyme of glycolysis, the up-regulation of GpmI accelerates the catalysis of the interconversion between 3-phosphoglycerate and 2-phosphoglycerate, whereas enolase (Eno) catalyzes the conversion of 2-phosphoglycerate into phosphoenolpyruvate (Yin et al., 2020). GpmI also plays an important role in the carbohydrate transport and metabolism. In addition, PGK not only is a glycolytic enzyme that plays an important role in the growth of biofilm, but also contributes to the formation of surface proteins. In biofilm formation, bacterial cells are embedded in the extracellular matrix, which can protect bacteria from a variety of environmental damages (Wang et al., 2016). Therefore, the tolerance of the strain could be effectively improved during the process of glycerol acclimation, so that the related proteins in the glycolysis pathway were up-regulated, and finally, the production yield of 1,3-PDO was increased. Concurrently, the multiple of down-regulated proteins, such as methylenetetrahydrofolate reductase (MetF), phosphoadenosine phosphosulfate reductase (CysH), 5-methyltetrahydropteroyltriglutamate-homocysteine *S*-methyltransferase (MetE), methionine adenosyltransferase (MetK) and so on, were down-regulated from 0.40 to 0.79. Commonly, a previous study has shown that high concentrations of homoserine are toxic to cells (Kingsbury and McCusker, 2010). In order to avoid the excessive accumulation of homoserine, decreased MetE, MetF, and MetK levels can inhibit met regulator. To prevent threonine biosynthesis, the up-regulation of ThrB can catalyze the over conversion of homoserine to *o*-phosphate-L-homoserine, which also can inhibit growth. This is a reversible transformation. When the concentration of homoserine decreased, *o*-phosphate-L-homoserine could be converted to homoserine to provide the precursor of methionine (Li et al., 2017). A dynamic balance is beneficial to the growth of cells and the synthesis of 1,3-PDO. For CysH, its down-regulation decrease cysteine (Longo et al., 2016), which reduces the effect of 1,3-PDO production.

**TABLE 1:** The enrichment analysis of the Gene Ontology (GO) and Kyoto Encyclopedia of Genes and Genomes (KEGG) pathway.

Function	Number of proteins	Log_2_(fold enrichment)
**GO term**		

**Up**		

**BP**		
Nucleoside monophosphate metabolic process	17	1.59
Phosphate-containing compound metabolic process	39	0.94
Phosphorylation	24	1.28
Phosphorus metabolic process	40	0.94
Ribonucleoside monophosphate metabolic process	17	1.66
Pyruvate metabolic process	10	1.91
Hexose metabolic process	9	2.11
Nucleoside diphosphate phosphorylation	9	1.98
Purine nucleoside diphosphate metabolic process	9	2.01
Purine ribonucleoside diphosphate metabolic process	9	2.01
Ribonucleoside diphosphate metabolic process	9	2.01
generation of precursor metabolites and energy	14	1.63
Purine ribonucleoside monophosphate metabolic process	14	1.60
Purine nucleoside monophosphate metabolic process	14	1.60
ATP generation from ADP	9	2.04
ADP metabolic process	9	2.04
Glycolytic process	9	2.04
Ribose phosphate metabolic process	18	1.24
Ribonucleotide metabolic process	17	1.36
Purine nucleotide metabolic process	15	1.36
Phosphotransferase activity, alcohol group as acceptor	14	1.46
**MF**		
Phosphotransferase activity, alcohol group as acceptor	14	1.46

**Down**		

**BP**		
Sulfur compound metabolic process	25	1.39
Sulfur compound biosynthetic process	23	1.44
sulfur amino acid biosynthetic process	13	1.82
Sulfur amino acid metabolic process	13	1.76
Methionine biosynthetic process	9	1.93
Methionine metabolic process	9	1.93
**MF**		
None		

**KEGG pathway**		

**Pathway**	**Number of proteins**	**Log_2_(fold enrichment)**

**Up**		

Methane metabolism	11	1.86
Glycolysis/gluconeogenesis	16	1.88
Fructose and mannose metabolism	7	2.10

**Down**		

Cysteine and methionine metabolism	16	1.46
Sulfur metabolism	12	1.64
Selenocompound metabolism	7	1.90

### The Analysis for Kyoto Encyclopedia of Genes and Genomes Pathway

**FIGURE 5:**
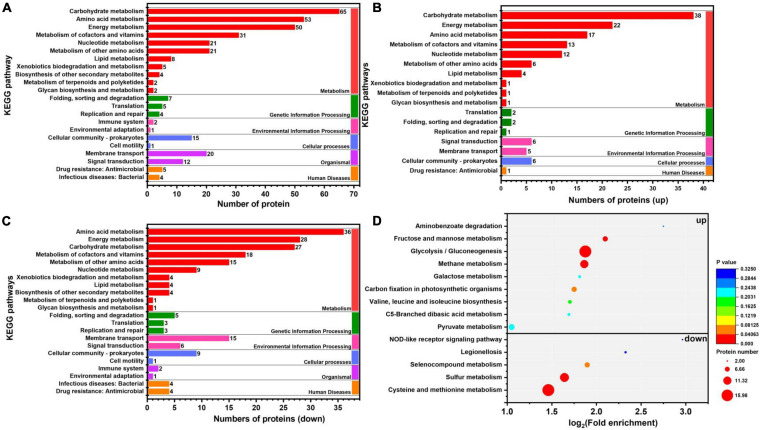
Kyoto Encyclopedia of Genes and Genomes (KEGG) pathway and enrichment analysis of G40 and G120–20 (+betaine). **(A)** KEGG pathway classification of differential protein. **(B)** The enrichment analysis of the KEGG pathway (up proteins). **(C)** The enrichment analysis of the KEGG pathway (down proteins). **(D)** The enrichment analysis of the KEGG pathway (up and down proteins). [The *p*-value (corrected) < 0.05].

**FIGURE 6:**
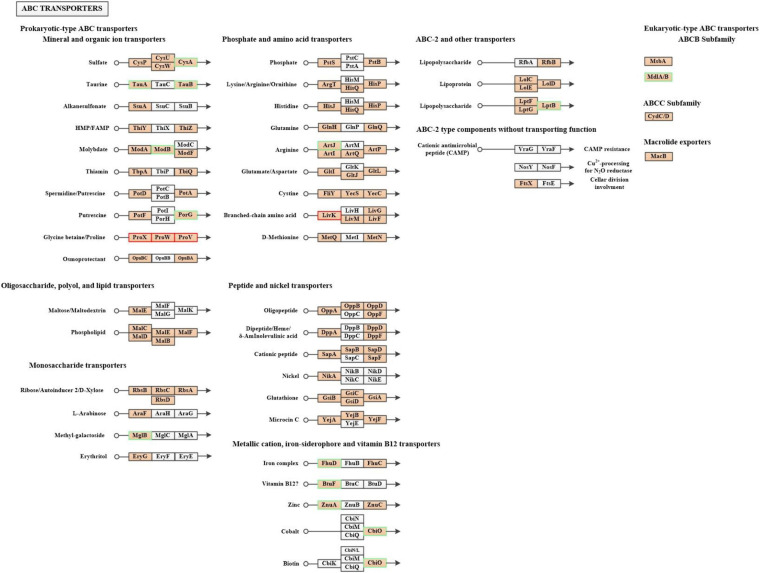
The ABC transporters of the G40 is compared with the G120–20 (+betaine) in KEGG pathway map. Proteins in orange block belong to the 3,284 proteins and in white block belonged to the undetected sample. Up-regulated proteins are marked by red, whereas down-regulated proteins are marked by green in 461 differential proteins. (The abbreviations for full name of all proteins are given in Supplementary Table 3).

In organisms, different gene products perform different biological functions through an orderly coordination. Therefore, the pathway information in the KEGG database helped us to understand the biological function of genes at the system level in *K. pneumoniae* (Jia et al., 2021). Figure 5A shows the KEGG pathway for differential proteins. The proteins were classified and annotated as follows: metabolism, genetic information processing, environmental information processing, cellular process, organismal systems, and human diseases. In this study, carbohydrate metabolism, amino acid metabolism, and energy metabolism were the most DEPs annotated in metabolism, with 65, 53, and 50 proteins, respectively. Combining with Figures 5B,C, there were 38 up-regulated proteins and 27 down-regulated proteins in carbohydrate metabolism. Simultaneously, in genetic information processing, the most DEPs were annotated in (translation), (folding, sorting, and degradation), and (replication and repair); in environmental information processing, the most DEPs were annotated in Immune system and environmental adaptation; in cellular processes, the most DEPs were annotated in cellular community–prokaryotes and cell motility. Despite the superfluous proteins, it required to investigate the KEGG pathway enrichment analysis. As shown in Figure 5D and Table 1, up-regulated proteins were linked to the methane metabolism, glycolysis/gluconeogenesis, fructose, and mannose metabolism. By contrast, the down-regulated proteins were involved in cysteine and methionine metabolism, sulfur metabolism, and selenocompound metabolism. Ultimately, there were 17 up-regulated proteins and 21 down-regulated proteins after further analysis. Therefore, further analysis was needed through the chord diagram of KEGG pathway enrichment. As can be seen from Figure 4B, the up-regulated proteins (glycolysis/gluconeogenesis, methane metabolism, fructose and mannose metabolism, carbon fixation in photosynthetic organisms, taurine and hypotaurine metabolism, pyruvate metabolism, propanoate metabolism, glyoxylate, and dicarboxylate metabolism, inositol phosphate metabolism) are more than down-regulated proteins, indicating that these KEGG pathways were activated. It suggested that the domesticated strain had an improvement in the production of 1,3-PDO compared to the predomesticated strain. As the degree of acclimation enhanced, the production of 1,3-PDO also increased, corresponding to the results in Figure 3. Therefore, the changes in the proteins of the metabolic pathway that produce 1,3-PDO needed to be further analyzed. In addition, the up-regulated and down-regulated proteins were similar to the analysis identified by GO terms, except that taurine import ATP-binding protein (TauA) and taurine-binding periplasmic protein (TauB) decreased, with the FC ranging from 0.35 to 0.43. As these two proteins were related to ABC transporters, further analysis of ABC transporters was given in Figure 6, illustrating that 17 differential proteins had changed. The CysA, TauA, TauB, ModB, PorG, MglB, ArtJ, FhuD, BtuF, ZnuA, CbiO, and LptB

were down-regulated, whereas the ProX, ProW, ProV, and Livk were up-regulated. ProV and ProW were membrane-associated proteins, and ProV had a considerable sequence identity with ATP-binding proteins from other periplasmic systems. ProX encoded the periplasmic glycine betaine-binding protein (Stirling et al., 1989; May et al., 2010). The biggest FC among them was ProX, which reached 4.39, revealing that betaine played an important role in fermentation.

When Na_2_CO_3_ was used to adjust the pH in the fermentation process, salt stress appeared with the continuous increase in Na_2_CO_3_. The osmotic pressure induced by the Na^+^ salt increased gradually, which resulted in the outflow of water, the loss of cell swelling pressure, and the change of solute concentration and cell volume (Fan et al., 2018). Compared with Ca(OH)_2_ used in commercialization as a pH-neutralizing agent (Wang et al., 2017; Liang et al., 2018), the output of 1,3-PDO was greatly affected. After the use of betaine, it was found that the yields were significantly improved as shown in Figure 1D. Betaine can be used not only as a stress protector or a stabilizer of intracellular enzymes to resist stress conditions, but also as a methyl donor for methylation. It can be accumulated at high concentrations (through transport or biosynthesis) in the cell to balance the osmotic pressure inside and outside the cell. On the other hand, it can increase the cell growth rate and improve the fermentation performance of the strain under high osmotic stress (high concentration of carbohydrate substrate or product) (Fan et al., 2018). In the 1,3-PDO fermentation process of *K. pneumoniae*, the stress of the Na^+^ salt as a neutralizer Na_2_CO_3_ and the yield of 1,3-PDO were improved by betaine, which offers an alternative way for the industrial pH neutralizer to avoid producing solid pollutants and thus greatly alleviating subsequent sewage treatment and environmental pollution.

### Analysis of Several Important Proteins in Metabolic Pathway

**FIGURE 7:**
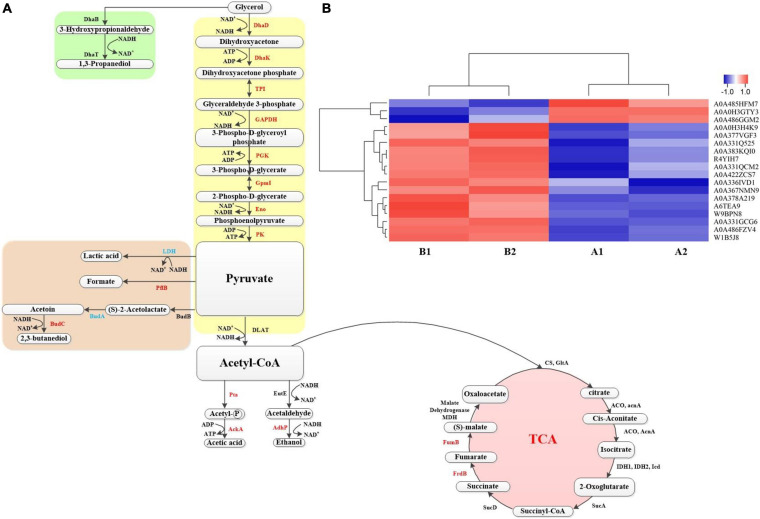
**(A)** The metabolic pathway for production of 1,3-propanediol by *K. pneumoniae*. The red represents the up-proteins, and the blue represents the down proteins, whereas the black represents the no change. **(B)** Heat map of proteins on the metabolic pathway. Each column in the figure represents a sample, and each row represents a protein. The color in the figure represents the relative expression amount of the protein in the group of samples; red represents the high expression amount of the protein in the sample, and blue represents the low expression amount. For the specific change trend of the expression amount, see the number label under the color bar at the top left.

In the above, some proteins that play a role in the production of 1,3-PDO by *K. pneumoniae* with the GO and KEGG pathway analysis and the important role of betaine in the fermentation process were analyzed However, protein changes of G120–20 compared with G40 in the metabolic pathway of *K. pneumoniae* producing 1,3-PDO still need further investigation. Combined with KEGG pathway analysis, the metabolic pathway of glycerol in *K. pneumoniae* is shown in Figure 7A: bacterial formation pathway, reduction pathway, and oxidation pathway. In the bacterial formation pathway, ATP was consumed, whereas ADP was produced in metabolism. In the reduction pathway: first, glycerol was converted to 3-hydroxyglyceraldehyde by DhaB and then converted to 1,3-PDO by DhaT. In G120–20 g/L (+betaine). Usually, 1,3-PDO oxidoreductase (DhaT) catalyzes the conversion of 3-hydroxypropionaldehyde (3-HPA) to 1,3-PDO, which is a key enzyme in the preparation of 1,3-PDO from glycerol. But DhaT is seriously inactivated by 3-HPA due to the reaction of 3-HPA with the sulfhydryl group of cysteine residue (Li et al., 2016). Although the DhaB and DhaT were not differential proteins in this article, they still had an effect on the production of 1,3-PDO by *K. pneumoniae*.

The oxidation pathway was similar to the glycolysis pathway, in that it generated ATP and reduced NADH_2_, which was required for bacterial growth. NADH_2_ was consumed during the 3-HPA-mediated production of 1,3-PDO, which was produced in the oxidation pathway. The oxidation pathway was mainly divided into three phases.

First, glycerol is converted into dihydroxyacetone through DhaD (NAD^+^ is required as a coenzyme to produce NADH_2_). Dihydroxyacetone is phosphorylated into glycolysis under the action of Dhak. As shown in Figure 7A and Table 2, DhaD and DhaK were up-regulated, and their FCs were 1.38 and 1.30, respectively. According to the report, DhaD encodes glycerol dehydrogenase in *K. pneumoniae* (Tang et al., 1982), and glycerol dehydrogenase and 1,3-propylene glycol oxidoreductase are the key enzymes for the conversion of glycerol to 1,3-PDO (Zhao et al., 2009). Therefore, the up-regulation of DhaD was conducive to the production of 1,3-PDO. It has been reported (Raynaud et al., 2011) that the DhaK, DhaL, and DhaM were belong to the PEP-dependent dihydroxyacetone kinases. It can be seen from Supplementary Table 3, DhaK [A6TEA9 contains dihydroxyacetone-binding sites (Gutknecht et al., 2001)], DhaL [A0A378G569 contains ADP-binding sites (Gutknecht et al., 2001)] and DhaM [A0A486FQT7, a phosphohistidine protein that can transfer phosphoryl groups from a phosphoryl carrier protein of the phosphotransferase system (HPr or enzyme I) to the DhaL-ADP complex (Gutknecht et al., 2001; Bachler et al., 2005)] were also up-regulated, indicating that dihydroxyacetone kinases were up-regulated. Glycerol dehydratase, 1,3-PDO oxidoreductase, glycerol dehydrogenase, and dihydroxyacetone kinase are encoded by an operon named dha, and their expression was consistent (Forage and Lin, 1982). It can be seen from Figures 7A,B; both were up-regulated at the same time, which also explains that the domesticated strain favors to produce 1,3-PDO.

**TABLE 2:** The protein of the metabolic pathway for *K. pneumoniae* producing 1,3-PDO.

Accession	Protein name	Description	FC	Log_2_FC	*P*	Sum up	Sum down
**Reduction pathway**							

W1DMB2	DhaB	Glycerol dehydratase reactivation factor large subunit	1.49	0.58	0.2364	0	0
Q7WRJ3	DhaT	1,3-Ppropanediol oxidoreductase	1.17	0.23	0.2874	0	0

**Oxidation pathway**							

A0A367NMN9	DhaD	Glycerol dehydrogenase	1.38	0.47	0.02303	1	0
A6TEA9	DhaK	Dihydroxyacetone kinase	1.30	0.38	0.03098	1	0
A0A0H3H4K9	TPI, tpiA	Triosephosphate isomerase	1.27	0.34	0.03373	1	0
A0A331Q525	GAPDH, GapA	NAD-dependent glyceraldehyde-3-phosphate dehydrogenase	3.03	1.60	0.02229	1	0
W1B5J8	PGK	Phosphoglycerate kinase	1.68	0.75	0.00423	1	0
A0A377VGF3	GpmI	2,3-bisphosphoglycerate-independent phosphoglycerate mutase	1.72	0.78	0.04665	1	0
R4YIH7	Eno	Enolase	1.23	0.30	0.01977	1	0
W9BPN8	PK, Pyk	Pyruvate kinase	1.48	0.57	0.02773	1	0
R4Y5U7	DLAT, AceF, PdhC	Acetyltransferase component of pyruvate dehydrogenase complex	0.35	−1.525	0.04124	0	0

**Lactate pathway**							

A0A485HFM7	LldD	L-lactate dehydrogenase	0.41	−1.27	0.04810	0	1
A0A486GGM2	LDH	L-lactate dehydrogenase	0.82	−0.28	0.04928	0	1

**2,3-butanediol pathway**							

A0A378G331	BudB	Acetolactate synthase	–	–	–	0	0
A0A0H3GTY3	BudA	Acetolactate decarboxylase	0.41	−1.27	0.00448	0	1
A0A422ZCS7	BudC	Butanediol dehydrogenase	1.83	0.87	0.02125	1	0

**Formic acid pathway**							

A0A383KQI0	PflB, PflD	Pyruvate formate-lyase	1.65	0.72	0.02091	1	0

**Acetic acid pathway**							

A0A336IVD1	Pta	Phosphate acetyltransferase	1.56	0.64	0.04410	1	0
A0A378A219	AckA	Acetate kinase	1.54	0.63	0.01629	1	0

**Ethanol pathway**							

A0A377VJU1	EutE	Acetaldehyde dehydrogenase	–	–	–	0	0
A0A331GCG6	AdhP	Alcohol dehydrogenase	1.72	0.78	0.00237	1	0

**TCA cycle pathway**							

A0A170J878	CS, GltA	Citrate synthase	–	–	–	0	0
A0A377ZR87	ACO, AcnA	Aconitate hydratase	–	–	–	0	0
A0A483EZU0	IDH1, IDH2, Icd	Isocitrate dehydrogenase [NADP]	–	–	–	0	0
A0A377 × 309	OGDH, SucA	2-Oxoglutarate dehydrogenase E1 component	–	–	–	0	0
A0A486KFH1	SucD	Succinate—CoA ligase [ADP-forming] subunit alpha	–	–	–	0	0
A0A486FZV4	FrdB	Succinate dehydrogenase iron-sulfur subunit	1.42	0.50	0.00340	1	0
A0A331QCM2	FumB	Fumarate hydratase class I	2.49	1.32	0.02712	1	0
A0A2S6E360	MDH	Malate dehydrogenase (Fragment)	–	–	–	0	0

Second, the dihydroxyacetone phosphate was further oxidized to pyruvate. In this process, triosephosphate isomerase (TPI), GAPDH, PGK, GpmI, Eno, and pyruvate kinase (PK) were up-regulated, and their corresponding FCs were 1.27, 3.03, 1.68, 1.72, 1.23, and 1.48, respectively. TPI plays a vital role in metabolism and is the key to efficient energy production (Zheng et al., 2006). The dihydroxyacetone phosphate was transformed into glyceraldehyde 3-phosphate through TPI. Because of the up-regulation of TPI, the accumulation of dihydroxyacetone phosphate can be reduced, as its toxicity would affect cell growth and survival (Kang et al., 2014). GAPDH catalyzes the conversion of glyceraldehyde 3-phosphate to glycerol 1,3-diphosphate and reduces NAD^+^ to NADH. Moreover, the up-regulation of GAPDH can shorten the fermentation time and inhibit the accumulation of some harmful by-products (such as lactic acid) (Yang et al., 2013). By comparing the protein changes in the two cases, most of the proteins in the glycolysis pathway were up-regulated, which helped to provide ATP and NADH for bacteria, promote the growth of the bacteria, and finally increase the yield of 1,3-PDO.

In addition, the by-products of pyruvate metabolism were lactic acid, formic acid, and 2,3-butanediol. The strain domestication may increase in yields of both the main product 1,3-PDO and by-products, so BudC and PflB were up-regulated. AckA and AdhP were also up-regulated when acetyl CoA produced by-products, such as ethanol and acetic acid. LDH and LldD encode L-lactate dehydrogenase (Aguilera et al., 2008; Fu et al., 2016). The down-regulation of lactate dehydrogenase reduces both the consumption of NADH and the formation of the by-product lactic acid and finally improves the output of 1,3-PDO (Xu et al., 2009).

The third step was that pyruvate could be further transferred to produce acetyl-CoA, and acetyl-CoA could enter the tricarboxylic acid (TCA) cycle to produce other small molecular substances. In this process, some energy was consumed, but there was a regeneration process of force reduction. In the whole TCA cycle, FumB and FrdB provide energy for bacteria. Therefore, the up-regulation of FumB and FrdB might accelerate cell growth and increase the yield of 1,3-PDO (Tseng et al., 2001; Xue et al., 2010; Huang et al., 2013; Li et al., 2019).

For the domesticated bacteria, most of the proteins in the metabolic process were up-regulated, resulting in a significant increase in the yield of 1,3-PDO. On the other hand, the 1,3-PDO yield could be further improved by the following genetic modifications, for example, the overexpression of some up-regulated genes (such as *dhaD*, *dhaK*, *tpi*, *gapA*, etc.), blocking the by-product pathway (such as knocking out *budC* to reduce the competition of by-products) and weakening *ldh* to reduce the consumption of NADH.

## Conclusion

The fermentation capabilities of 1,3-PDO by glycerol domesticated strains at the concentrations of 40, 60, 80, 100, 120, and 140 g/L were compared. It was found that the strain domesticated with 120 g/L glycerol had the highest capability to produce 1,3-PDO, reaching 59.41 g/L. To further improve the yield of 1,3-PDO, the strain domesticated with 120–20 g/L glycerol concentration (*K. pneumoniae* x546) was obtained, and the yield reached 69.35 g/L. In addition, in order to overcome the osmotic pressure problem caused by excessive Na^+^ in the fermentation system, betaine was added to the fermentation medium, making the yield further increase to 74.44 g/L and shortening the fermentation time from 40 to 24 h. Based on TMT, it was found that regulating genes, such as *dhaD*, *dhak*, *budC*, *ldh*, and so on, were able to enhance the yield of 1,3-PDO. Moreover, the introduction of Na_2_CO_3_ and betaine in the fermentation process will render the formation of 1,3-PDO more environment-friendly and facilitate industrial adoption of this technology in the future.

## Data Availability Statement

The original contributions presented in the study are publicly available. This data can be found here: PRIDE database Project Name: *Klebsiella pneumoniae* x546, ATCC15380, TMT Project accession: PXD028396.

## Author Contributions

XW carried out the experimental work, analyzed the data, and wrote the manuscript. HC and PW performed the data analysis and participated in the manuscript editing and revise. JJ and LZ helped to partial experiment and figure processing. JX helped to edit the manuscript and involved in discussion in the manuscript preparation. JW was responsible for the experiment design and supervision. All authors read and approved the final manuscript.

## Conflict of Interest

The authors declare that the research was conducted in the absence of any commercial or financial relationships that could be construed as a potential conflict of interest.

## Publisher’s Note

All claims expressed in this article are solely those of the authors and do not necessarily represent those of their affiliated organizations, or those of the publisher, the editors and the reviewers. Any product that may be evaluated in this article, or claim that may be made by its manufacturer, is not guaranteed or endorsed by the publisher.

## Footnotes

1
www.uniprot.org/taxonomy/?query=ATCC15380&sort=score

2
http://geneontology.org/

3
http://www.genome.jp/kegg/
