# Supplementary material for: Rational Proteomic Analysis of a New Domesticated Klebsiella pneumoniae x546 Producing 1,3-Propanediol
Source: Front Microbiol. 2021 Nov 26;12:770109. doi: 10.3389/fmicb.2021.770109 (PMC8662357; doi:10.3389/fmicb.2021.770109)
Supplement: Supplementary file 2 [file Data_Sheet_1.docx]

**Additional file 1**

Rational proteomic analysis of a new domesticated *Klebsiella pneumoniae* x546 producing 1,3-propanediol

Xin Wang^1,2,4,5^, Lin Zhang^3^, Hong Chen^1,2^, Pan Wang^1,2^, Ying Yin^1,2^, Jiaqi Jin^1,2^, Jianwei Xu^4,5^ and Jianping Wen^1,2^*

^1^ Key Laboratory of Systems Bioengineering (Ministry of Education), Tianjin University, Tianjin 300072, P. R. China

^2^ SynBio Research Platform, Collaborative Innovation Center of Chemical Science and Engineering (Tianjin), School of Chemical Engineering and Technology, Tianjin University, Tianjin 300072, P. R. China

^3^ Dalian Petrochemical Research Institute of Sinopec, Dalian 116000, P. R. China

^4^ Institute of Materials Research and Engineering, 2 Fusionopolis Way, Agency for Science, Technology and Research, Singapore 138634

^5^ Department of Chemistry, National University of Singapore, 3 Science Drive 3, Singapore 117543

* Correspondence author: Jianping Wen

Telephone: +86-022-27892061;

Fax: +86-022-27892061;

E-mail: jpwen@tju.edu.cn

**The adaptive laboratory evolution of *K. pneumoniae* x546**

Firstly, this is a step-by-step domestication process. The *K. pneumoniae* ATCC 15380 was activated at a glycerol concentration of 40 g/L, then rises with a glycerol concentration gradient of 20 g/L until the domestication experiment at a glycerol concentration of 140 g/L. At this process, the *K. pneumoniae* domesticated with 40 g/L (60, 80, 100, 120, 140 g/L) glycerol could be obtained. Secondly, the strain was domesticated with a concentration of 120 g/L glycerol and then returned to 20 g/L glycerol for domestication. Therefore, the strain x546 could be achieved.

All strains were domesticated in shake flask. Take the domestication process from the concentration of 40 g/L to 60 g/L glycerol as an example:

Firstly, the acclimated strain with glycerol concentration of 40 g/L was selected from the solid medium with glycerol concentration of 40 g/L, and added into a 250 mL flask with 100 mL seed medium (glycerol concentration of 60 g/L. Refer to the method section in the paper for other medium components). The optimum conditions are initial medium pH 7.0, culture temperature 37℃, and rotational speed of shaker 150 rpm/min. After 9.0~9.5 h, the OD_600_ will become 2.0~2.5 (as shown in Fig. S1). Then, the bacterial solution was diluted and coated on the solid medium at the concentration of 60 g/L glycerol. The strain grew up after 12~14 h (as shown in Fig. S2).

Then, a new round of repeated acclimation experiment was carried out in the seed medium at the concentration of 60 g/L glycerol, and the experimental cycle took more than half a month. Until the strain grew well on solid medium. At this time, the strain domestication experiment with glycerol concentration of 60 g/L was completed. The next round of strain domestication experiment from 60 g/L to 80 g/L glycerol concentration can be carried out.


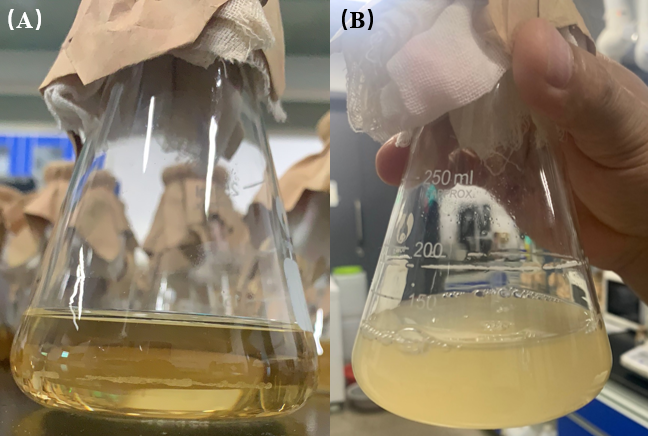


**Fig. S1 (A)Status of strain in seed medium at 0 h. (B)Status of strain in seed medium at 9.5 h.**


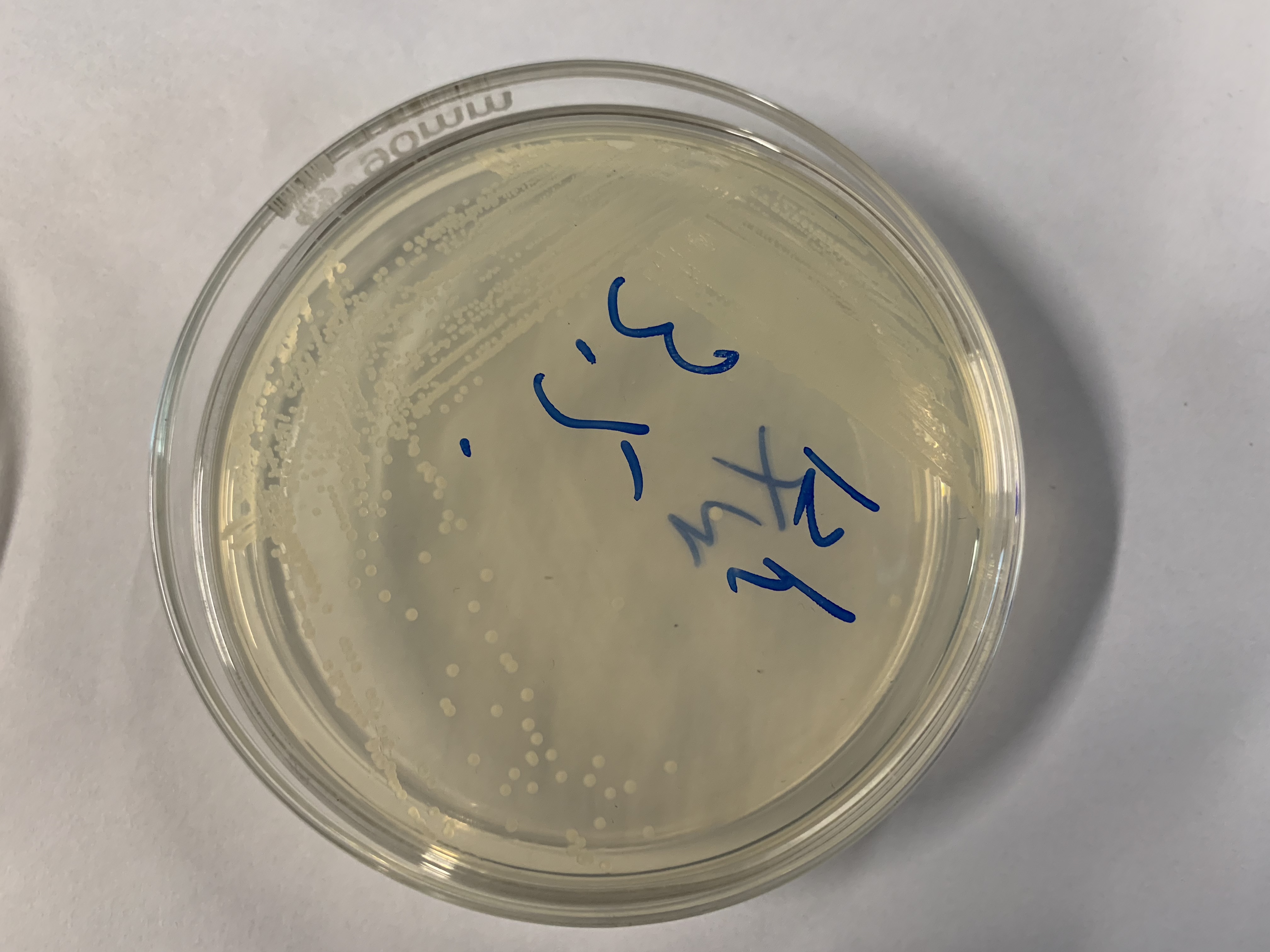


**Fig. S2 Status of strain on solid medium at 12 h.**


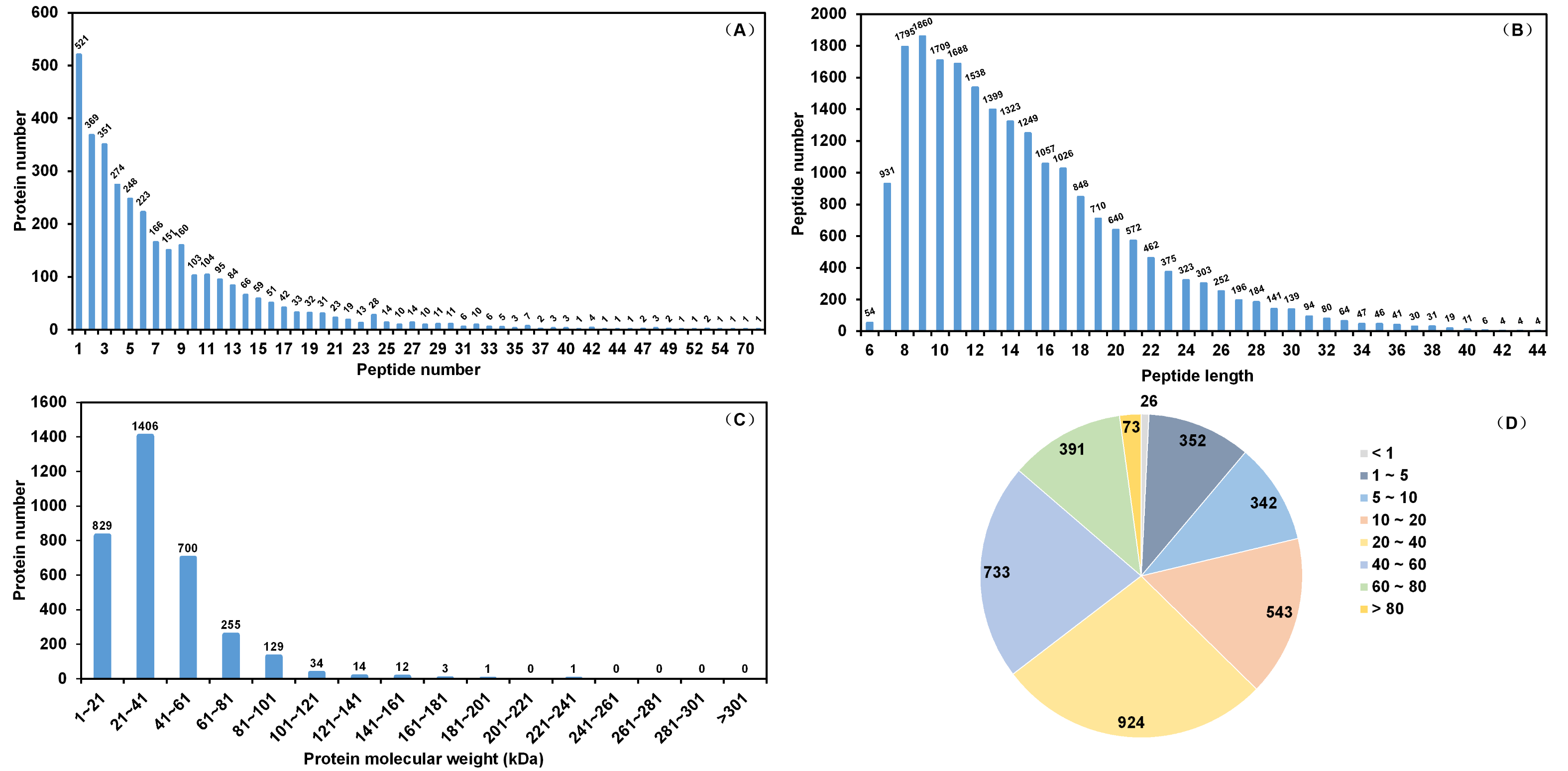


**Fig. S3 (A) Number distribution of peptides contained in identified proteins.**

**(B) The length distribution of the identified peptides.**

**(C)** **The molecular weight distribution of the identified protein.**

**(D) Coverage distribution of identified proteins**

**Quantitative Real-Time PCR (qRT-PCR) Analysis**

The experiment was conducted under the same glycerol environment: the first factor was the difference of strains, which were the strains acclimated with 40 g/L glycerol concentration and the strains acclimated with 120-20 g/L glycerol concentration. There were some differences between the two domesticated strains. The second factor was the effect of betaine.

**Verify the differentially expressed proteins by qRT-PCR**

In order to verify the accuracy of proteomic data, qRT-PCR was used to verify some differential proteins identified and quantified by TMT. qRT-PCR were carried out on samples at 10 h between the G40 and G120-20, G120-20 and G120-20 (+betaine).

(1) Total RNA extraction: the total RNA of the bacteria was extracted by using the RNA prepPure culture cell/bacterial total RNA extraction kit of Tiangen company according to the operation instructions, and treated with DNase I to remove the residual DNA fragments. The RNA samples were quantified by spectrophotometer at 260 nm and 280 nm, and purified by 1% formaldehyde agarose gel electrophoresis.

(2) cDNA synthesis: the synthesis of cDNA was completed by using the fast quant cDNA first strand synthesis kit of Tiangen company according to the operation instructions.

PCR reaction: according to the genome sequence of *Klebsiella pneumoniae* NCTC 418 included in GenBank in NCBI database. The synthetic primers of the target gene were designed by primer design software (Primer Premier 5.0). The primer sequences were shown in Table S1. Taking the 16S rRNA gene of *Klebsiella pneumoniae* as the internal standard control, qRT-PCR was performed with Real Master Mix (SYBRGreen) kit of Tiangen company according to the operation instructions. Relative quantitation 2^-ΔΔCT^ method was used to analyze the qRT-PCR data. To calculate the CT value obtained from the experiment, and the relative differences of gene transcription were obtained.

**Table S1 Sequences of primer pairs for qRT-PCR**

| **Primer name** | **Sequence 5'-3'** |
| --- | --- |
| DhaD-RTF | CCACGCAATCCACAACG |
| DhaD-RTR | TGGCAGAAGCCCAGCAC |
| DhaK-RTF | GGTGGCTGAGATGGCGATTC |
| DhaK-RTR | ACGGCACGGCGTTGATAG |
| TPI-RTF | GAAATGTATCTGGACCTGGCTAA |
| TPI-RTR | CGTCGGATTCTTTGTGGTAG |
| PGK-RTF | TCGTTGGTGGTTCTAAAGTG |
| PGK-RTR | TTCGTACAGGGATTTACCG |
| GpmI-RTF | TCGCCAACCCGTTACTGA |
| GpmI-RTR | CACCATCGCCATGATGTGA |
| PK-RTF | AAGGCTTCGCAGAACCAA |
| PK-RTR | GCCGCAATCTCACGCACT |
| ProW-RTF | CTGTTCGGCATCGGCAAC |
| ProW-RTR | CCGCAGGCACCTGGTTTATC |
| ProV-RTF | GCGAAGCATCAACGCACTA |
| ProV-RTR | TTTACGAATCAGGCCAACC |
| ProX-RTF | CCCATAACCAGGGCAACT |
| ProX-RTR | CGGAAGGGAGGAGAACG |
| LldD-RTF | ATCGCCACTTTCATCTCTTTCTC |
| LldD-RTR | CTACTGGGCCGCGCTTAC |
| BudA-RTF | CGCTGGTTAAAGCGGAACAC |
| BudA-RTR | ATCGACCAGCAAATCCCCTC |
| CysA-RTF | GCACTATGCGCTGTTCCG |
| CysA-RTR | TGGTCACTTTGGCTTTAATCG |
| TauA-RTF | AGGACTTCGCCGAGCAGCAT |
| TauA-RTR | TTCCCGCCGCAGGCACTTTG |

**To verify the differentially expressed proteins by qRT-PCR transcription level**

In order to verify the reliability of the proteomic data, the following 13 proteins were selected for verification. The qRT-PCR method was used to verify the transcription level, including 9 up-regulated proteins and 4 down-regulated proteins. The comparison of data between the relative transcription abundance obtained by qRT-PCR and the relative expression abundance obtained by the TMT group of the corresponding protein was shown in Table S2. It could be seen from Table S2 that the qRT-PCR data and the proteomic data had a high degree of positive correlation. The significant positive correlation between TMT and qRT-PCR results indicates the reliability of the TMT proteomic analysis data.

**Table S2 Comparison of ratios calculated from TMT proteomics and qRT-PCR analysis**

| **Protein name** | **Proteomic ratio**  **(G40 and**  **G120-20 (+betaine))** | **qRT-PCR ratio**  **(G40 and G120-20)** | **qRT-PCR ratio**  **(G120-20 and**  **G120-20 (+betaine))** | **Description** |
| --- | --- | --- | --- | --- |
| DhaD | 1.38 ± 0.100 | 1.57 ± 0.043 | 1.015 ± 0.072 | Glycerol dehydrogenase |
| DhaK | 1.30 ± 0.052 | 1.46 ± 0.054 | 1.004 ± 0.005 | Dihydroxyacetone kinase |
| TPI | 1.27 ± 0.017 | 1.58 ± 0.038 | 1.001 ± 0.046 | Triosephosphate isomerase |
| PGK | 1.68 ± 0.082 | 1.80 ± 0.021 | 1.010 ± 0.146 | Phosphoglycerate kinase |
| GpmI | 1.72 ± 0.115 | 1.25 ± 0.071 | 1.002 ± 0.074 | 2,3-bisphosphoglycerate-independent phosphoglycerate mutase |
| PK | 1.48 ± 0.056 | 1.52 ± 0.115 | 1.027 ± 0.176 | Pyruvate kinase |
| ProW | 1.30 ± 0.084 | 1.002 ± 0.051 | 2.31 ± 0.142 | L-proline glycine betaine ABC transport system permease protein |
| ProV | 3.03 ± 0.153 | 1.015 ± 0.047 | 4.37 ± 0.153 | L-proline glycine betaine ABC transport system permease |
| ProX | 4.65 ± 1.004 | 1.007 ± 0.254 | 5.36 ± 0.543 | L-proline glycine betaine binding ABC transporter protein |
| LldD | 0.42 ± 0.019 | 0.35 ± 0.021 | 0.98 ± 0.023 | L-lactate dehydrogenase |
| BudA | 0.41 ± 0.039 | 0.47 ± 0.053 | 0.99 ± 0.042 | Acetolactate decarboxylase |
| CysA | 0.76 ± 0.028 | 0.98 ± 0.024 | 0.73 ± 0.051 | Sulfate/thiosulfate import ATP-binding protein |
| TauA | 0.44 ± 0.023 | 0.97± 0.019 | 0.37 ± 0.016 | Taurine import ATP-binding protein |

It could be seen from the data in the Table S2 that when the fermentation conditions were the same and the G40 and G120-20 were compared, DhaD, DhaK, TPI and so on are positively correlated. While ProW, ProV and ProX did not change significantly. When only for G120-20, with or without betaine, it was found that the qRT-PCR data of ProW, ProV, ProX, etc. were positively correlated with proteomic data, and there were no significant changes in others. It showed the reliability of proteomic analysis data.
